# Genome-wide identification and analysis of glyceraldehyde-3-phosphate dehydrogenase family reveals the role of GmGAPDH14 to improve salt tolerance in soybean (*Glycine max* L.)

**DOI:** 10.3389/fpls.2023.1193044

**Published:** 2023-06-06

**Authors:** Xunchao Zhao, Jie Wang, Ning Xia, Yuewen Qu, Yuhang Zhan, Weili Teng, Haiyan Li, Wenbin Li, Yongguang Li, Xue Zhao, Yingpeng Han

**Affiliations:** Key Laboratory of Soybean Biology in Chinese Ministry of Education (Key Laboratory of Soybean Biology and Breeding/Genetics of Chinese Agriculture Ministry), Northeast Agricultural University, Harbin, China

**Keywords:** GAPDH protein, hairy roots, salt tolerance, soybean, gene family

## Abstract

**Introduction:**

Glyceraldehyde-3-phosphate dehydrogenase (GAPDH) is an essential key enzyme in the glycolytic pathway and plays an important role in stress responses. Although GAPDH family genes have been found in different plant species, the determination of their gene family analysis and their functional roles in soybean are still unknown.

**Methods:**

In this study, gene sequence and expression data were obtained using online tools, and systematic evolution, expression profile analysis, and qRT-PCR analysis were conducted.

**Results and Discussion:**

Here a total of 16 GmGAPDH genes were identified on nine chromosomes, which were classified into three clusters. Additionally, all GmGAPDH genes harbor two highly conserved domains, including Gp_dh_N (PF00044) and Gp_dh_C (PF02800). The qRTPCR analysis also showed that most GmGAPDH genes significantly responded to multiple abiotic stresses, including NaHCO3, polyethylene glycol, cold, and salt. Among them, GmGAPDH14 was extraordinarily induced by salt stress. The GmGAPDH14 gene was cloned and overexpressed through soybean hair roots. The overexpressed transgenic soybean plants of the GmGAPDH14 gene have also shown better growth than that of control plants. Moreover, the overexpressed transgenic plants of GmGAPDH14 gene had higher activities of superoxide dismutase but lower malonaldehyde (MDA) content than those of control plants under salt stress. Meanwhile, a total of four haplotypes were found for the GmGAPDH14 gene, and haplotypes 2, 3, and 4 were beneficial for the tolerance of soybean to salt stress. These results suggest that the GmGAPDH14 gene might be involved in the process of soybean tolerance to salt stress. The results of this study will be valuable in understanding the role of GAPDH genes in the abiotic stress response of soybean.

## Introduction

Glyceraldehyde-3-phosphate dehydrogenase (GAPDH) is a key enzyme in the glycolytic metabolic pathway, which widely exists in biological cells (Zhang et al., 2019). GAPDHs catalyzed glyceraldehyde-3-phosphate to form 1,3-biphosphoglycerate in the presence of NAD^+^ and inorganic phosphate (Sirover, 2011). The major functions of the GAPDH gene refer to immune response (Henry et al., 2015), expression regulation (Zhang et al., 2017), and autophagy (Colell et al., 2007).

In plants, *GAPDH* genes are involved in glycolytic or photosynthetic pathways (Plaxton, 1996). Meanwhile, *GAPDH* genes can be divided into three categories according to their functions in cells (Guo et al., 2012; Guo et al., 2014). In chloroplasts, NADP-specific GAPDHs (GAPA/B) were involved in photosynthetic CO_2_ fixation. In the cytoplasm, NAD-dependent GAPDH (GAPC) converted glyceraldehyde-3-P (Ga3P) to 1,3-bisphosphoglycerate. In plastids, GAPCp isoforms may be involved in glycolytic energy production. Moreover, all GAPDH proteins contained highly conserved domains, including the Gp_dh_N (PF00044) and Gp_dh_C (PF02800) domains (Jiao et al., 2011; Zeng et al., 2016; Miao et al., 2019). It was also found that some GAPDHs also contained CP12 (PF02672) domain.

To date, a series of *GAPDH* genes have been cloned and characterized, including *Arabidopsis thaliana* (Guo et al., 2014), *Oryza sativa* (Lim et al., 2021), *Zea may* (Bustos et al., 2007), and *Cucumis sativus* (Chaturvedi et al., 2016). Based on subcellular localization, it has been proven that GAPDH was divided into cytosolic (Cy) and plastic (P) isoforms (Miao et al., 2019; Wei et al., 2022). In *A. thaliana*, GAPDH genes distributed in different subcellular compartments: GAPC1 and GAPC2 were located in the cytosol, and the rest of the GAPDH genes were located in plastids (Rius et al., 2008; Anoman et al., 2015). Some researchers have revealed that GAPCs can regulate the accumulation of oil content in seeds (Guo et al., 2014). The seed oil content was reduced by 3% when *GAPDH* was knocked out of the cytoplasm in *A. thaliana*, suggesting that cytosolic *GAPDH* was vital for regulating the content of seed oil (Guo et al., 2014). Furthermore, the plastidic GAPCp has been shown to be involved in starch metabolism (Muñoz-Bertomeu et al., 2009). In soybean, the knockdown of *GAPC1* decreased the nodule nitrogenase activity without affecting the nodule weight (Ke et al., 2022). Moreover, the key role of *GAPDH* genes in plant growth and development and responses to abiotic stresses has been extensively confirmed, including heat (Kim et al., 2020), cold (Liu et al., 2017), salinity (Cho et al., 2014), and drought (Li et al., 2019). Previous studies have shown that the overexpression of *PsGAPDH* can increased salt tolerance in potato (Jeong et al., 2001). In *Arabidopsis*, the overexpression of *TaGApC* gene from Chinese spring *Triticum aestivum* displayed improved drought tolerance by decreasing the reactive oxygen species (ROS) levels (Zhang et al., 2020). Furthermore, it was also found that salicylic acid restrains the GAPDH activity *in vitro* (Pokotylo et al., 2020).

Soybean was the main oil crop in the world (Holle and Damme, 2015). However, the yield and the quality of soybean were often affected by abiotic stresses such as low temperature, drought, and salinization (Feng et al., 2020). Therefore, it was significant to study the salt resistance mechanism of soybean and excavate the stress-resistant genes for improving the yield and quality of soybean. Although *GAPDHs* have been characterized and analyzed in many plant species, the characterization of the *GAPDH* gene family in soybean is still limited, and it is unknown how *GAPDH* regulates the molecular mechanism of salt stress in soybean.

Although most studies have described the biological and physiological functions of the *GAPDH* gene, few research were known in terms of the functional divergence of the *GAPDH* gene family in soybean. In this study, 16 of the *GAPDH* gene members in soybean were identified, and their phylogenetic relationships, gene structure, chromosomal localization, and stress responses were analyzed. Furthermore, the function of *GmGAPDH14* gene in soybean tolerance to salt stress was tentatively verified, indicating the important role of *GmGAPDH14* gene in salt stress.

## Materials and methods

### Identification of the GAPDH gene family in soybean

To identify the *GAPDH* gene sequence of soybean, systematic BLASTP was conducted against the soybean reference genome database (https://www.soybase.org/) and the Phytozome database (https://phytozome-next.jgi.doe.gov/) using the published *Arabidopsis* GAPDH as alignment sequence. The screening threshold was set to E-value (<10^−10^), and the protein length was greater than 200 aa. The candidate *GAPDH* genes were determined by SMART (http://smart.embl-heidelberg.de) and Pfam (http://pfam.xfam.org/) software with both Gp_dh_N and Gp_dh_C domains. The open reading frame length was obtained from the Phytozome database. The molecular weight and the isoelectric point values were downloaded from the ExPASy (Artimo et al., 2012) software (https://web.expasy.org/protparam/). CELLO 2.5 (Yu et al., 2004) was used to predict subcellular localization.

### Phylogeny, gene structure, and conserved domain analysis

The protein sequences of GAPDHs from soybean (*Glycine max*), maize (*Zea mays*), rice (*O. sativa*), and *Arabidopsis* (*A. thaliana*) were used to construct a phylogenetic tree using the neighbor-joining method and bootstrap test set at 1,000 replications through the MEGA7.0 software (Kumar et al., 2016). The exon/intron structures of *GmGAPDHs* were demonstrated at the GSDS online server (Hu et al., 2015). The coding and genomic sequences of *GmGAPDH* were collected from the Phytozome database. The conserved domains of GAPDH were determined by SMART (http://smart.embl-heidelberg.de) (Letunic et al., 2015) and Pfam (http://pfam.xfam.org/) (Finn et al., 2016) software, including Gp_dh_N and Gp_dh_C domains, and the structure of GAPDH proteins was visualized using the IBS 6.0 software (Liu et al., 2015).

### Promoter analysis of *GmGAPDHs*

To investigate the critical *cis*-acting elements in the promoter of *GmGAPDH* genes, the sequence at 2.0 kb upstream of the position of the ATG codon in these genes was obtained from the Phytozome database (https://phytozome-next.jgi.doe.gov/).The plant CARE database was used to predict the *cis*-acting regulatory elements, including motifs related to plant growth and development, plant hormone responses, and abiotic and biotic stress responses.

### Expression analysis of *GmGAPDHs* during soybean development and response to abiotic stresses

The expression patterns of soybean *GmGAPDHs* at different tissues were obtained using the Phytozome database. The heat maps were generated by cluster analysis with the TBtools software (Chen et al., 2020), and the expression data were log_2_-transformed. To explore the expression patterns of *GmGAPDHs* in seeds at different developmental stages, soybean seeds (DN50) were collected at 10, 20, 30, and 40 days after flowering (DAF), and the total RNA extraction of each sample was performed to analyze the expression patterns of *GmGAPDHs* under abiotic stresses, including cold, salt, NaHCO_3_, and drought. Briefly, soybeans (DN50) were grown in a plant incubator. There were two different plant cultivation methods used: (a) for low temperature treatment, seeds of soybean were sown in soil and vermiculite (v:v/1:1) and (b) for the salinity, NaHCO_3_, and drought treatments, soybeans were grown in a hydroponic culture, and the growth condition was 24°C and a 16-h/8-h (day/night) daily photoperiod cycle. Second-trifoliolate-stage seedlings of uniform growth were subjected to cold treatment with a low temperature of 4°C, salt treatment with 150 mM salt, drought treatment with 20% polyethylene glycol (PEG, 6,000 g/M), and alkali treatment with 100 mM NaHCO_3._ The soybean leaves were sampled at 0, 6, 12, and 24 h after the treatments. The sample total RNA was extracted using Trizol reagent (Invitrogen). The expressions of *GmGAPDH* genes in soybean seed samples at 10 DAF were used as a calibrator. The expressions of *GmGAPDH* genes in soybean samples at 0 h were used as a calibrator. *GmACTIN4* (GenBank accession no. AF049106) was used as an internal reference. Quantitative real-time RT-PCR (qRT-PCR) was conducted using the CFX Connect TMreal-time system (BIO-RAD) with SYBR Select Master Mix RT-PCR (SYBR Green, TOYOBO, Osaka, Japan). Three biological replicates with three technical replicates were applied to each sample. The expression levels of *GmGAPDHs* were calculated using the 2^–ΔΔct^ method (Hong et al., 2010), and all primers used for the expression analysis were listed in **Supplementary Table S1**.

### *Agrobacterium*-mediated transformation of *GmGAPDH14* soybean hairy roots

Soybean cultivar DN50 was used for the *Agrobacterium rhizogenes* strain K599 transformation in soybean hypocotyls. The cDNA of *GmGAPDH14* was directly ligated into the vector *pCambia3300*. The recombinant plasmid and empty *pCambia3300* vector (EV) were transferred into *Agrobacterium rhizogenes* strain K599 and then injected into the hypocotyls following a previous report (Kereszt et al., 2007; Yu et al., 2021). The transgenic plants were identified by PCR amplification, and the non-transgenic hairy roots in the seedlings were removed.

### Detection of physiological indicators after salt treatments

The hairy root soybean plants were grown in Hoagland nutrient solution, and the growth chamber was set at a 16/8-h light–dark daily photoperiodic cycle. The transgenic plants were treated with 0 and 150 mM salt for 3 days (d), respectively.

The leaves of overexpression *GmGAPDH14* (OE-*GmGAPDH14*) and EV seedlings were analyzed to measure physiological indicators. The measurements of superoxide dismutase (SOD) and malonaldehyde (MDA) were conducted according to corresponding assay kit protocols (Cominbio, Suzhou, China). All measurements were obtained with three biological replicates.

### Prediction and haplotype analysis of *GmGAPDH14* gene for soybean salt resistance

A total of 131 soybean germplasms were collected and grown in Harbin for 2 consecutive years (2019–2020). All accessions were treated with 0 and 150 mM salt. The relative swelling rate was obtained according to Zhang’s method (Zhang et al., 2014). According to genomic re-sequencing data, the SNPs in genomic regions including the promoter, 5′UTRs, exon, intron, and 3′UTRs of *GmGAPDH14* gene were analyzed in 131 soybean lines using the generalized linear model (GLM) method as conducted with Tassel version 5.0 software (Bradbury et al., 2017).

### Statistical analysis

The statistical significance was evaluated using Student’s *t*-test as performed with the SPSS 22.0 software. The significance levels were *p < 0.05 and **p < 0.01. The standard deviation (mean ± SD) was calculated with at least three biological replicates.

## Results

### Characterization of the GAPDH gene families of soybean

To identify the *GAPDH* gene family of soybean, *GAPDH* genes were selected by BLASTp tool with *Arabidopsis* as the alignment sequence. A total of 16 *GAPDH* genes (*GmGAPDH1*–*GmGAPDH16*) were retrieved (**Supplementary Table S2**). The full-length CDS sequences of *GmGAPDH1*–*GmGAPDH16* varied from 786 to 1,362 bp. The isoelectric points of GmGAPDHs ranged from 6.54 to 8.71, and the molecular weight of GmGAPDHs ranged from 28.2 to 48.4 kDa (**Supplementary Table S2**).

**Figure 1:**
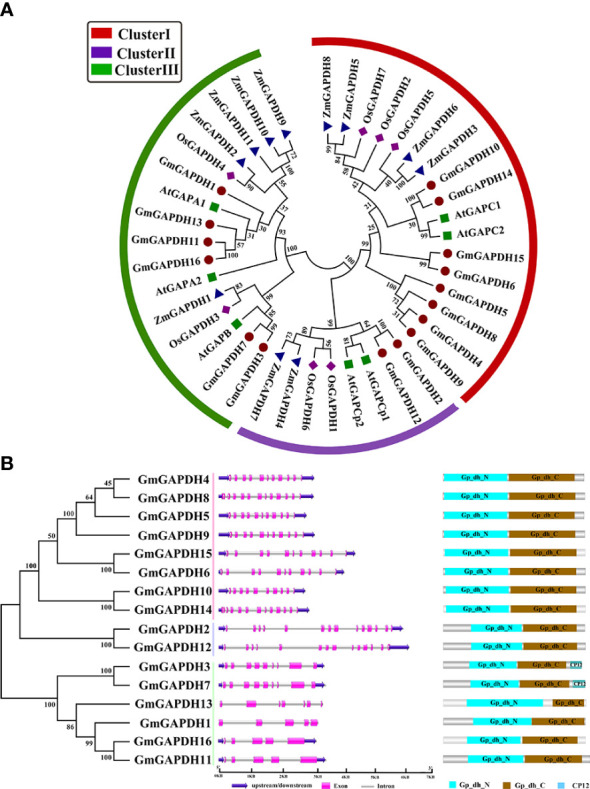
**(A)** Phylogenetic tree construction of GAPDH proteins from soybean (*G. max*), maize (*Z. mays*), *Arabidopsis* (*A. thaliana*), and rice (*O. sativa*). The phylogenetic tree was constructed through the neighbor-joining method based on MEGA7.0. The different colors of the rings represent different subfamilies: red, blue, and green represent clusters I, II, and III, respectively. **(B)** Exon–intron structure and domain analysis of GmGAPDHs of soybean. The untranslated region, exon, and intron are represented with blue, pink, and gray, respectively. Different-colored boxes were represented using different signal peptides.

To understand the evolutionary relationship of GmGAPDHs in soybean, the amino acid sequences of GmGAPDHs from *A. thaliana* (seven), *Z. mays* (12), and *O. sativa* (seven) were obtained from the NCBI database (https://www.ncbi.nlm.nih.gov/), and a phylogenetic tree was built. The phylogenetic tree indicated that the GAPDH proteins of soybean were clearly divided into three clusters (I–III) (**Figure 1A**). Cluster I, consisting of GmGAPDH4–6, GmGAPDH8–10, and GmGAPDH14–15, corresponded with cytosol isoforms containing AtGAPC proteins. Cluster II, including GmGAPDH2 and GmGAPDH12, corresponded with plastid isoforms containing AtGAPCp1 and AtGAPCp2 proteins. Cluster III, covering GmGAPDH1/3/7/11/13/16, corresponded with AtGAPA1, AtGAPA2, and AtGAPB proteins (**Figure 1A**).

### Exon/intron structure and the conserved domain of soybean *GAPDH* genes

The structures of soybean *GAPDH* genes were characterized with the GSDS software. As shown in **Figure 1**, cluster II (*GmGAPDH2* and *GmGAPDH12*) had the largest number of exons, including 12 exons. The exon number of cluster I ranged between 10 (*GmGAPDH10*) and 12 (*GmGAPDH14*); the remaining *GmGAPDHs* contained nine exons. The exon number of cluster III ranged from five to nine, and only *GmGAPDH3*/*7* had nine exons; the remaining *GmGAPDHs* had five exons (**Figure 1B**).

The conserved domains analysis for 16 GmGAPDHs indicated that the GmGAPDHs revealed a multiple-domain protein, including Gp_dh_N (PF00044) and Gp_dh_C (PF02800) domains (**Figure 1B**). The Gp_dh_N domain (INGFGRIGR) and Gp_dh_C (GAAKAV) sequences were identified as highly conserved in the GmGAPDHs (**Supplementary Figures S1**, **S2**). A conserved active site (PS00071: ASCTTNCL) was found in most GmGAPDHs, except for GmGAPDH1 and GmGAPDH13 (**Supplementary Figure S1**). The similarity of the gene structure and conserved domains of soybean GAPDH genes implies that they have undergone gene duplication during evolution.

### Analysis of regulatory elements in the promoter of *GmGAPDHs*

**Figure 2:**
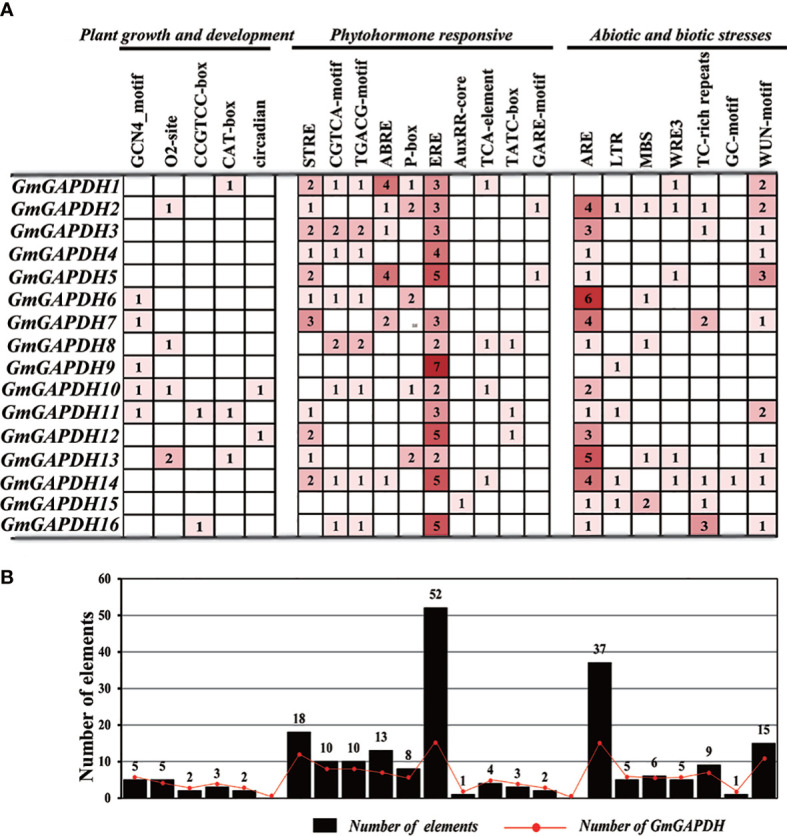
Analysis of cis-elements in the promoter of *GAPDH* genes. **(A)** Number of cis-elements in the 2.0-kb promoter region upstream of *GmGAPDH* genes. **(B)** Statistical analysis for the total number of *GmGAPDH* genes; the black box corresponds to the total number of cis-elements, and the red dot corresponds to the number of *GmGAPDH* genes.

To obtain the *cis*-elements of *GmGAPDHs*, sequencing of 2,000 bp upstream of all *GmGAPDHs* gene was performed based on the PlantCARE software. As shown in **Figure 2A**, a total of 22 *cis*-elements were found with plant growth and development, phytohormone-responsive, and abiotic and biotic stresses in the upstreams of 16 *GmGAPDH* genes. The ERE and ARE *cis*-elements were found with almost all *GmGAPDH* genes. The GCN4_motif (endosperm expression) elements were discovered in *GmGAPDH6*-*7* and *GmGAPDH9*-*11*. Meanwhile, *GmGAPDH15* and *GmGAPDH14* were analyzed only involving AuxRR-core (auxin-responsive) and GC-motif (anoxic-specific inducibility) elements, respectively. It was noteworthy that there were five *GmGAPDHs* that harbored low temperature responsiveness (LTR) elements while five *GmGAPDHs* contained MBS (drought-responsive) elements. In addition, O_2_ site (gliadin metabolic regulatory), CCGTCC box (specific activation), and CAT box (meristem expression) were found in the *GmGAPDHs* gene. TC-rich repeats (defense and stress responsiveness), GC motif (involved in anoxic-specific inducibility), and WUN motif (mechanical injury response) elements were observed in six, one, and 10 *GmGAPDH* genes, respectively (**Figure 2B**). These results showed that the GAPDH family may play an important role in growth and development and response to environmental stress in soybean.

### Synteny analysis of *GmGAPDHs*

**Figure 3:**
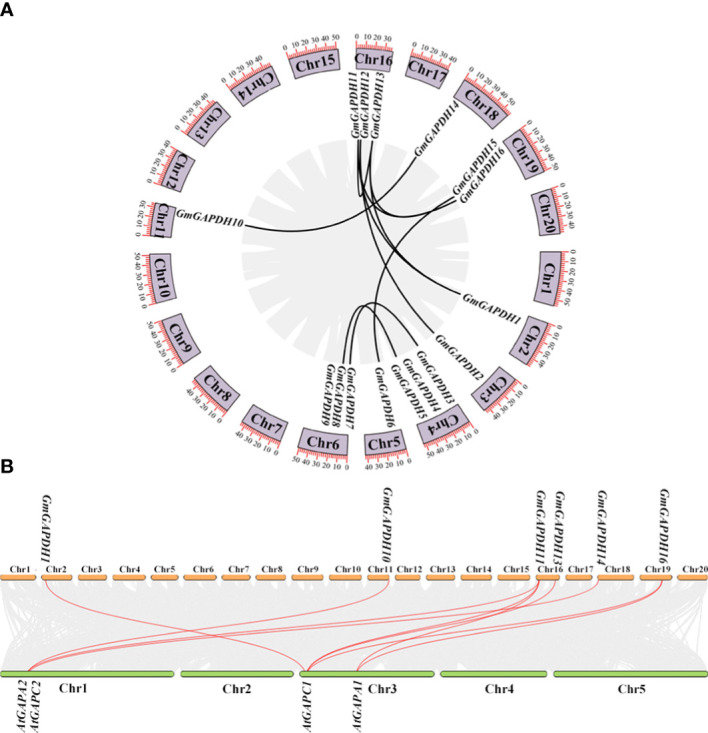
Syntenic analysis of *GmGAPDH* family genes. **(A)** Chromosome location and duplication of *GmGAPDH* genes on soybean genome. **(B)** Syntenic analysis of *GAPDHs* with the corresponding genes in *G max* and *A thaliana*.

To further characterize duplicated events within the soybean genome, a synteny analysis of *GmGAPDH* genes was performed. As shown in **Figure 3A**, the *GmGAPDH* genes were scattered on nine of the 20 soybean chromosomes. The nine soybean chromosomes distributed one to three *GmGAPDH* genes (**Figure 3A**). The replication relationship with the soybean *GAPDH* genes was analyzed. A total of 10 duplicated gene pairs were identified within the soybean (**Figure 3A**). Meanwhile, a synteny analysis was conducted from *G. max* and *A. thaliana*. As shown in **Figure 3B**, *GmGAPDHs* had a replication relationship with *AtGAPDHs*, including nine replication relationship pairs between *G. max* and *A. thaliana* (*GmGAPDH1*/*AtGAPC1*, *GmGAPDH10*/*AtGAPC2*, *GmGAPDH11*/*AtGAPA2*, *GmGAPDH11*/*AtGAPA1*, *GmGAPDH11*/*AtGAPA2*, *GAPDH13*/*AtGAPC1*, *GAPDH14*/*AtGAPC2*, *GAPDH16*/*AtGAPA1*, and *GAPDH16*/*AtGAPC1*) (**Figure 3B**). The duplicated genes showed their common genomic origin and maybe functional similarity.

### Expression profiles of soybean GAPDHs in diverse tissues and developmental stages

**Figure 4:**
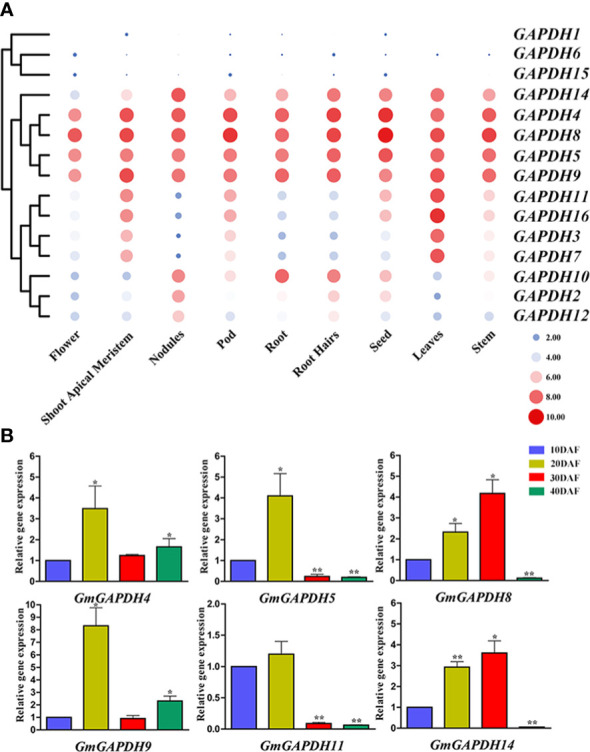
Expression levels of *GmGAPDH* genes during different tissue and developmental stages. **(A)** The expression profile analyses of soybean *GAPDH* genes were characterized in different tissues. The expressions of different tissues were displayed in heat maps. The color scale indicates the log2 expression level, the red circles indicate the high transcription levels, and the blue circles indicate the low transcription levels. **(B)** Relative expression level of *GmGAPDHs* in different developing seeds at 10, 20, 30, and 40 days after flowering in soybean (DN50). The expression of *GmGAPDHs* in developing seeds at 10 DAF was used as the internal reference. Student’s *t*-test was carried out to determine the significance levels (*P < 0.05, **P < 0.01).

To determine the expression pattern of soybean *GAPDH* genes in different development phases, we retrieved the high-throughput sequencing data of the Phytozome database and conducted an expression analysis. As demonstrated in **Figure 4**, the expression of *GmGAPDHs* was revealed in diverse tissues. *GmGAPDH4*, *5*, *8*, *9*, and *14* were found to have a higher expression in different tissues. Meanwhile, the *GmGAPDH1*, *GmGAPDH6*, and *GmGAPDH15* genes were feebly expressed in nine different tissues. Moreover, *GmGAPDH3*, *7*, *11*, and *16* were expressed only in the shoot apical meristem and leaf tissues. *GmGAPDH2* and *GmGAPDH12* were especially expressed only in nodules (**Figure 4A**). Moreover, the expression of *GmGAPDHs* during the development of soybean seed is shown in **Figure 4B** (10 to 40 DAF). The expression levels of *GmGAPDH4*, *GmGAPDH5*, *GmGAPDH9*, and *GmGAPDH11* were found to be upregulated in seeds at 20 DAF. The expression of the *GmGAPDH8* and *GmGAPDH14* genes was significantly upregulated in seeds at 30 DAF.

### Expression profiles of *GmGAPDH* genes under abiotic stresses

**Figure 5:**
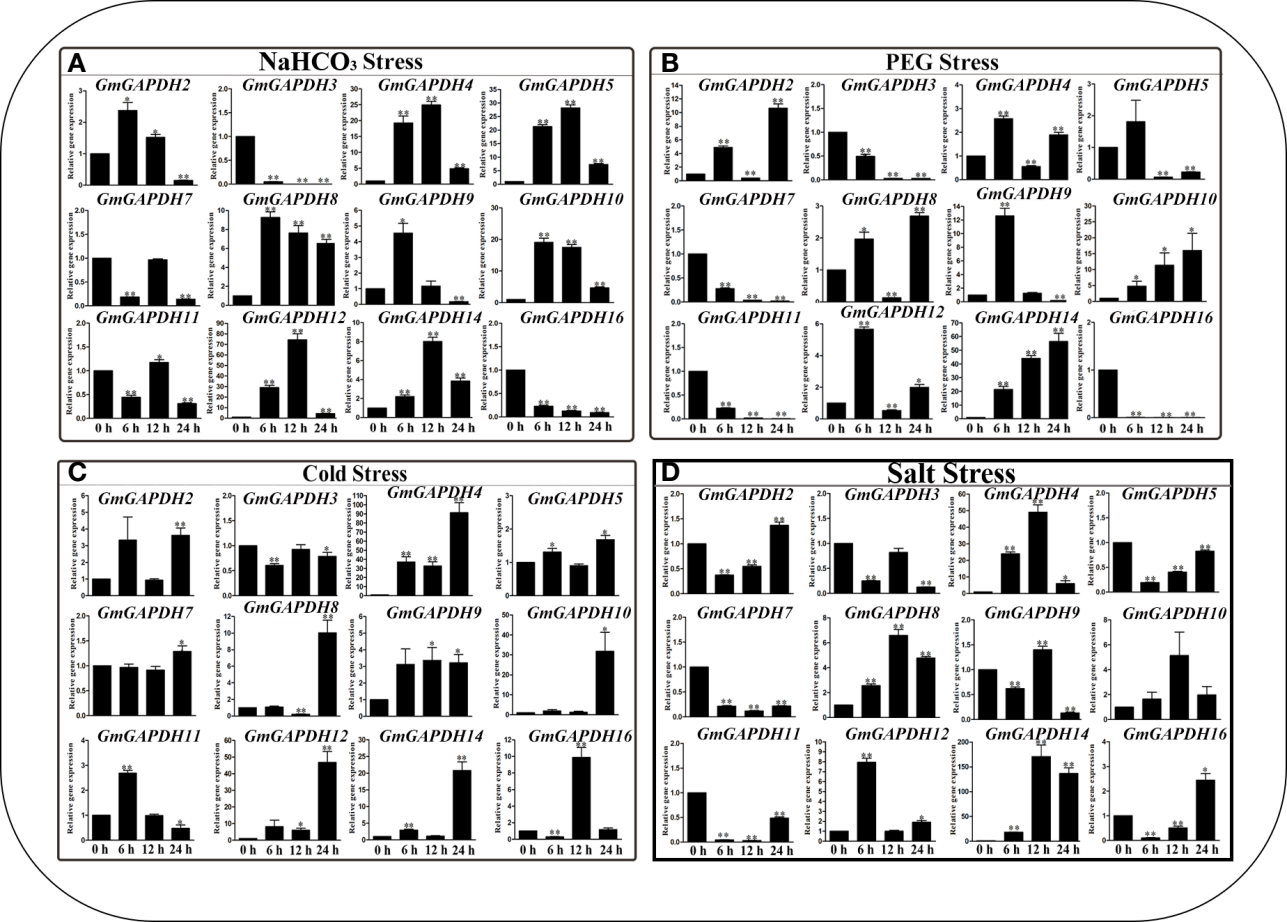
Expression level of *GmGAPDHs* in response to NaHCO_3_ **(A)**, polyethylene glycol **(B)**, cold **(C)**, and salt **(D)** stresses in the leaves of soybean. The expression of *GmGAPDHs* in a non-stress environment was used as a calibrator. Three technical replicates and three biological replicates were applied to each sample. The expression level of *GmGAPDHs* was calculated with the 2^–ΔΔct^ method.

To confirm the role of *GmGAPDH* gene responses to various abiotic stresses, the transcription level of 16 *GmGAPDH* genes under NaHCO_3_ (100 mM), PEG (20%), Cold (4°C), and Slat (150 mM) stresses was determined by qRT-PCR. Under simulated alkali stress using NaHCO_3_, *GmGAPDH 4*, *5*, *8*, *10*, *12*, and *14* were extraordinarily upregulated (more than six folds) and peaked at 6 and 12 h, respectively (**Figure 5A**). After simulated drought stress using PEG, *GmGAPDH14* was upregulated by more than 20 folds in 24 h after the treatment. In comparison, the expression of *GmGAPDH16* significantly decreased in 6, 12, and 24 h, respectively (**Figure 5B**). In response to cold treatment, most of *GmGAPDHs* had upregulated expression, especially *GmGAPDH4*, which had a significantly higher level of expression at 24 h (**Figure 5C**). Furthermore, the *GmGAPDH* genes were shown to have a higher expression level at different timepoints of cold stress. The expression levels of *GmGAPDHs* were different under simulated salt stress with salt (**Figure 5D**). The *GmGAPDH14* gene was shown to have the highest expression level at 6, 12, and 24, which was upregulated by more than 150 folds than that at 24 h. Meanwhile, the *GmGAPDH4* gene exhibited a higher expression level at 6 and 12 h (20 and 40 folds). In contrast to other stresses, the whole expression of *GmGAPDHs* under NaHCO_3_ stress was found to be relatively low (**Figure 5A**). Remarkably, the expression of *GmGAPDH14* was sharply induced under salt stress, indicating that this gene might play a role in salt stress resistance.

### Overexpression of *GmGAPDH14* improved the tolerance to salt stress

To deeply illustrate how *GmGAPDH14* genes respond to salt stress, hairy roots with *Agrobacterium rhizogenes* K599 containing *pCambia3300*-*GmGAPDH14* plasmid or the *pCambia3300* empty vector were transformed. Eight soybeans were proven through the PCR method to be positive transgenic. Furthermore, it was found that the effect of overexpression of *GmGAPDH14* is such that it can regulate soybean hair roots in response to salt tolerance. Furthermore, 2-week soybean positive lines, involving 0 or 150 mM salt for 3 days, were transferred.

**Figure 6:**
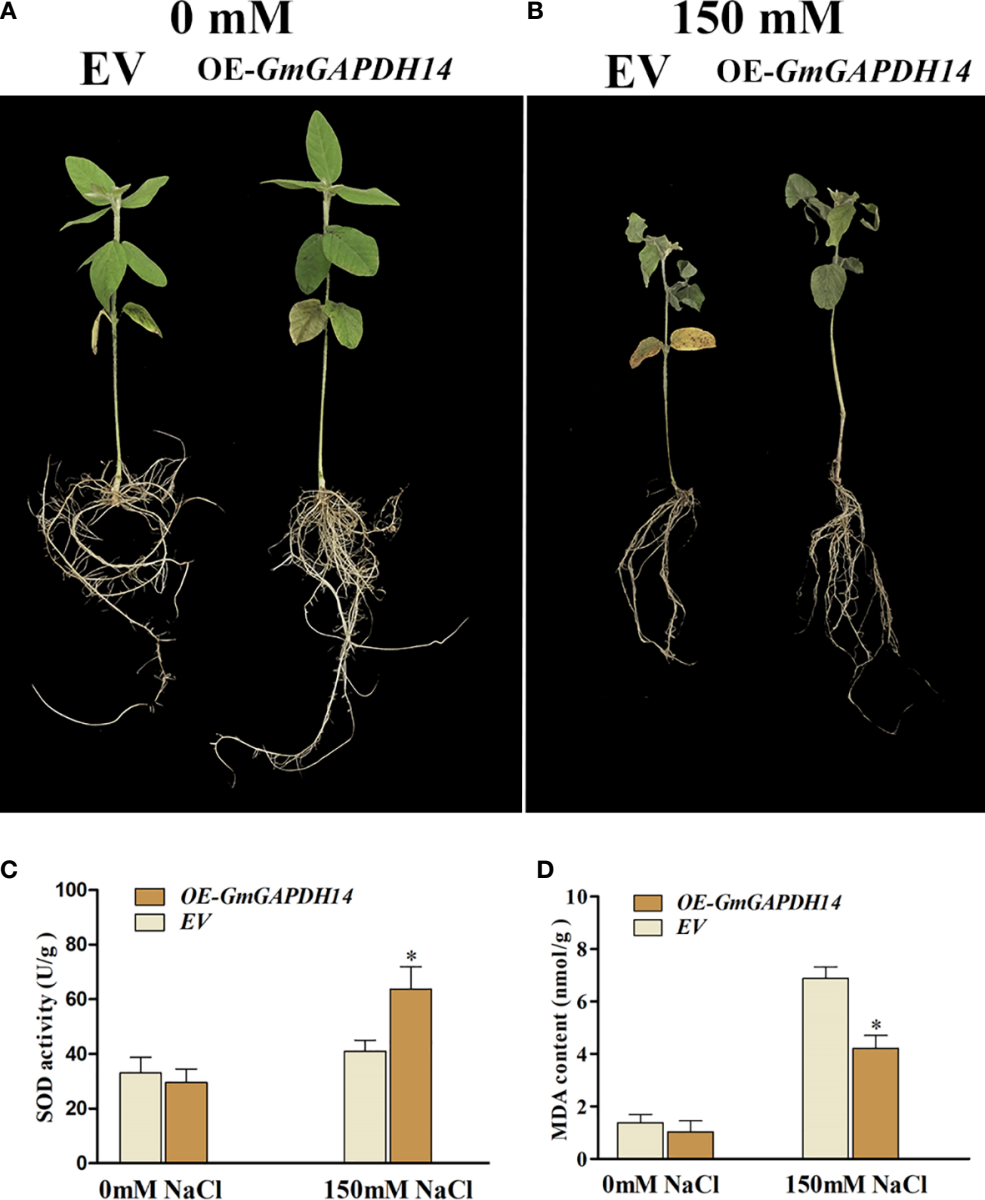
Effect of *GmGAPDH14* overexpression: **(A)** 0 mM salt, **(B)** 150 mM salt, **(C)** superoxide dismutase (SOD) activity, and **(D)** malondialdehyde (MDA) content in transgenic lines under salt stress. EV represents empty vector pCambia3300 (transgenic soybean hairy roots, control), and OE-GmGAPDH14 represents recombinant vector pCambia3300–*GmGAPDH14* (transgenic soybean hairy roots). The activity of SOD and the MDA content in soybean seedling at 3 days after 150 mM salt treatment. The asterisks represent significant differences between EV and OE-GmGAPDH14 by Student’s *t*-test (^∗^ *P* < 0.05).

Previous studies showed that plant *GAPDHs* are involved in functions such as response to oxidative stress (Guo et al., 2012). Hence, the overexpression of *GmGAPDH14* may further increase the antioxidant level under salt stress in this study. Therefore, the SOD activity and the MDA content were tested in soybeans positive transgenic at 3 d after 150 mM salt treatment. After 3 d of 150 mM salt stress, the overexpression of *GmGAPDH14* was exhibited to enhance the resistance to salt stress than those of the control (transformed by *pCambia3300* EV) (**Figures 6A, B**). As shown in **Figure 6**, the OE-*GmGAPDH14* lines had a higher activity of SOD than that of the EV lines (**Figure 6C**). The above-mentioned results showed that *GmGAPDH14* could participate in regulating the ROS level. The MDA content showed that the contents of the OE-*GmGAPDH14* lines were significantly lower than that of the EV lines after the salt treatment (**Figure 6D**).

### Haplotype analysis of *GmGAPDH14* gene for salt stress

**Figure 7:**
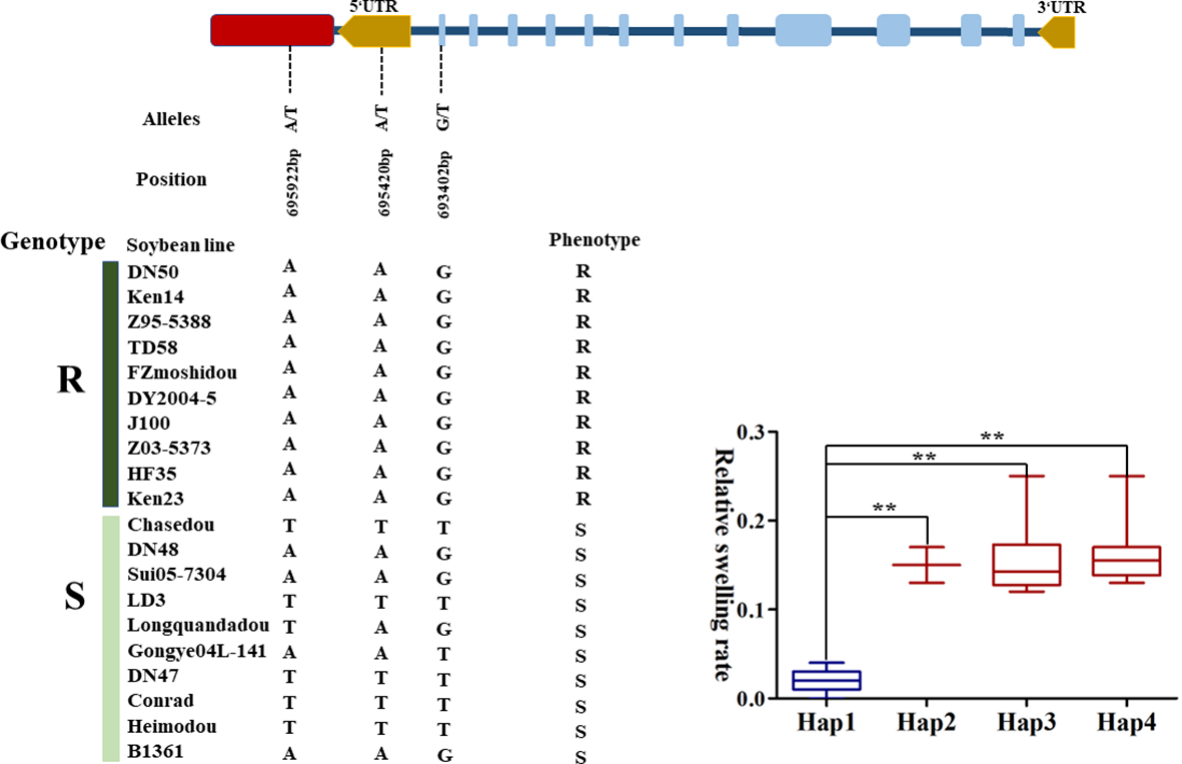
Haplotype analysis of *GmGAPDH14* genes related to salt stress. The asterisks represent significant differences by Student’s *t*-test (^∗∗^ *P* < 0.01).

To further confirm the potential effects of *GmGAPDH14* gene for salt stress, gene-based association analysis was applied through the GLM method. A total of five SNPs in the *GmGAPDH14* gene were identified among 131 lines (**Supplementary Table S3**). All the five SNPs were significantly associated with salt stress, and they were located in the exon, intron, UTR region, and upstream regions of the *GmGAPDH14* gene, respectively (**Table 1**). Four haplotypes of the *GmGAPDH14* gene were defined by the five SNPs. Haplotypes 2, 3, and 4 were composed of the combination of TAG, TTT, and AAT alleles and were beneficial for the salt tolerance of soybean. The carriers of haplotype 1 were composed of a combination of AAG alleles which tended to be sensitive to salt stress (**Figure 7**, **Supplementary Figure S3**). The difference of salt tolerance between the carriers of two haplotypes reached a very significant level.

**Table 1:** The association between SNP in *GmGAPDH14* gene and soybean resistance to salt based on 131 soybean germplasms.

Chromosome	Position (bp)	Region	Alleles	Year	-log10(p)
18	693402	Exon	G/T	2019	2.74
18	693891	Intron	C/T	2019	1.82
18	695147	Intron	T/C	2019	3.56
18	695420	UTR	A/T	2019	2.35
18	695922	Promoter	A/T	2019	2.74
18	693402	Exon	G/T	2020	1.08
18	693891	Intron	C/T	2020	2.6
18	695147	Intron	T/C	2020	2.14
18	695420	UTR	A/T	2020	1.53
18	695922	Promoter	A/T	2020	1.08

## Discussion

GAPDH is a pivotal enzyme in the glycolytic pathway. Previous studies have exhibited that GAPDHs play a crucial role in plant growth and response to various stresses (Guo et al., 2012; Liu et al., 2017; Kim et al., 2020). In this study, the candidate genes of soybean *GAPDH* family were identified, which were proven to regulate plant growth and stress. Although *GmGAPDH* genes have been analyzed in many plants, including *Arabidopsis* (Guo et al., 2014; Kim et al., 2020), potato (Liu et al., 2017), wheat (Li et al., 2019), and rice (Wang et al., 2021), nevertheless, finite information was found about the GAPDH molecular function of soybean.

In the present study, a total of 16 *GmGAPDH* genes were identified from the soybean genome. The phylogenetic analysis can clearly prove the evolutionary relationships between soybean GAPDH and those of other species. The result showed that 16 GAPDHs were segmented into three clusters based on their different subcellular locations. Previous studies showed that the different GAPDHs can target various regions (Rius et al., 2008; Muñoz-Bertomeu et al., 2009). For cluster I, eight GmGAPDHs isoforms were located on the cytosol of *Arabidopsis* GAPCs; for cluster II, two GmGAPDHs isoforms were located on the chloroplast; and for cluster III, six GmGAPDHs isoforms were located on the plastid (**Figure 1**) (Vescovi et al., 2013; Anoman et al., 2015; Marri et al., 2015). The result showed that the three distinct types of GmGAPDHs can implement corresponding functions in plants. Gene duplication was the key mechanism for the creation of unique evolutionary innovations, which mainly include segmental and tandem duplications (Jiao et al., 2011). The previous studies showed that a family gene will show a highly conserved duplication style in various species (Innan and Kondrashov, 2010). In this study, most of the *GmGAPDH* genes were found to carry out segmental duplication in the soybean genome (**Figure 3**). The result of this study indicated that soybean GAPDH did not maintain a conserved duplication. These duplicated genes proved a common genomic source and performed similar functions, and segmental duplication was advantageous to extend of *GAPDH* genes family in soybean.

In this study, to reveal the response of soybean *GAPDHs* to abiotic stresses, the expression pattern of *GAPDH* genes was tested *via* qRT-PCR analysis. The promoter sequences of the soybean *GAPDHs* involved a number of *cis*-elements—for example, ERE, ARE, and LTR (**Figure 2**). According to this result, NaHCO_3_, PEG, cold stress, and salt stress can significantly induce *GmGAPDHs* expression (**Figure 5**). *AtGAPC* was found to interact with phospholipase Dδ (PLDδ), transmit H_2_O_2_ signaling under drought stress, and increase the seed oil content (Guo et al., 2012; Kim et al., 2020). In wheat, *GAPDH12* was found to be remarkably upregulated under salt, cold, high temperature, and drought stresses (Zeng et al., 2016). The overexpression of *GAPC3* can improve salt tolerance in rice (Zhang et al., 2011). In *Arabidopsis*, the overexpression of *TaGAPC1* enhanced the tolerance to drought stress (Zhang et al., 2019). In potato, *StGAPC1*, *StGAPC2*, and *StGAPC3* were found to be cold-induced in the tubers (Liu et al., 2017). In *Arabidopsis*, the overexpression of GAPC improved its heat tolerance (Kim et al., 2020). In this study, *GAPDH12* was found to be strongly under NaHCO_3_ stress at 12 h. In PEG stress, *GAPDH14* was found to be strongly under stress at 24 h. In cold stress, *GAPDH4* was found to be strongly under stress at 24 h. It is worth noting that *GmGAPDH14* responded more strongly to salt stress than the other genes, and *GmGAPDH14* reached a maximum expression level with 12 h to salt stress (about 150 folds). Therefore, *GAPDH14* may play an important role in response to salt stress.

Previous studies showed that GAPDH proteins—PsGAPDH, NbGAPC, and AtGAPC—play key roles in growth and abiotic stress response in plants (Guo et al., 2014; Han et al., 2015; Lim et al., 2021). Next, to further understand the molecular function of *GmGAPDH14* in response to salt stress, the overexpression of GmGAPDH14 significantly increased the salt tolerance of transgenic soybean lines (**Figures 6A, B**). In this study, the SOD activity of OE-*GmGAPDH14* plants was significantly higher than that of EV plants. The above-mentioned data showed that *GmGAPDH14* may play an important role in reducing ROS accumulation under salt stress, which was consistent with the results of previous studies (Zhang et al., 2011). The content of MDA was commonly considered as a marker of oxidative stress (Anjum et al., 2015). It was certified that the EV plants were found to have more serious damage than OE-*GmGAPDH14* plants (**Figure 6D**). In addition, the five SNPs of the *GmGAPDH14* gene were significantly associated with soybean tolerance to salt stress. Four haplotypes of the *GmGAPDH14* gene were defined by the five SNPs, and the difference of salt tolerance between the carriers of the four haplotypes reached a very significant level. Meantime, the 693402 position SNP was found to be located in the Gp_dh_C domain. This result suggests that variations in the domain may enhance the salt tolerance of the *GAPDH14* gene. In this study, the involvement of *GmGAPDH14* gene in soybean salt tolerance was verified, and the salt tolerance effect of the gene related to the natural variation of the gene sequence was also proven. These results may provide ideas for exploring the beneficial salt-resistant SNPs.

In conclusion, a total of 16 soybean *GAPDH* genes were clearly divided into three clusters. These *GmGAPDH* genes had a different expression level under various abiotic stresses. The 16 *GmGAPDH* genes, including *GmGAPDH14*, had a markedly induced response to salinity.

## Data availability statement

The original contributions presented in the study are included in the article/**Supplementary Material**. Further inquiries can be directed to the corresponding authors.

## Author contributions

Conceptualization: YH, YL, and XueZ; methodology: XunZ, NX, and JW; software: YQ, YZ, and WT; formal analysis: XunZ and JW; investigation: XunZ, HL, and WL; resources: YH; data curation: YQ, JW, and YZ; writing—original: XunZ; writing—review and editing: YH and YL; supervision: YH and YL; funding acquisition: YH and XueZ. All authors All authors contributed to the article and approved the submitted version.

## Supplementary material

Supplementary Figure 1 Analysis of the conserved active site of GAPDH in soybean.

Supplementary Figure 2 Analysis of the conserved motifs of GAPDH in soybean.

Supplementary Figure 3 Haplotype analysis of GmGAPDH14 gene related to salt stress.
