# Supplementary material for: Genome-wide identification and analysis of glyceraldehyde-3-phosphate dehydrogenase family reveals the role of GmGAPDH14 to improve salt tolerance in soybean (Glycine max L.)
Source: Front Plant Sci. 2023 Jun 6;14:1193044. doi: 10.3389/fpls.2023.1193044 (PMC10281054; doi:10.3389/fpls.2023.1193044)
Supplement: Supplementary Figure 1 — Analysis of the conserved active site of GAPDH in soybean. [file DataSheet_1.zip › Supplementary Material/TableS2.docx]

| **Gene name** | **Gene ID^1^** | **Direction** | **ORF^2^ length (bp)** | **Isoelectric**  **point** | **Molecular Weight (KDa)** |
| --- | --- | --- | --- | --- | --- |
| *GAPDH1* | *Glyma.02G068800* | forward | 831 | 7.81 | 29.2 |
| *GAPDH2* | *Glyma.03G092700* | reverse | 1257 | 8.71 | 44.6 |
| *GAPDH3* | *Glyma.04G015900* | forward | 1356 | 7.10 | 48.1 |
| *GAPDH4* | *Glyma.04G193400* | forward | 1017 | 6.72 | 36.7 |
| *GAPDH5* | *Glyma.04G193500* | forward | 1017 | 7.10 | 36.8 |
| *GAPDH6* | *Glyma.05G073100* | reverse | 1014 | 8.18 | 36.4 |
| *GAPDH7* | *Glyma.06G015900* | forward | 1362 | 6.76 | 48.4 |
| *GAPDH8* | *Glyma.06G172600* | reverse | 1017 | 6.72 | 36.7 |
| *GAPDH9* | *Glyma.06G172700* | reverse | 1017 | 6.72 | 36.7 |
| *GAPDH10* | *Glyma.11G247600* | forward | 1023 | 6.98 | 37.0 |
| *GAPDH11* | *Glyma.16G044900* | forward | 1212 | 7.61 | 43.2 |
| *GAPDH12* | *Glyma.16G081400* | forward | 1257 | 8.83 | 44.8 |
| *GAPDH13* | *Glyma.16G150200* | forward | 786 | 7.55 | 28.2 |
| *GAPDH14* | *Glyma.18G009700* | reverse | 1023 | 6.97 | 36.9 |
| *GAPDH15* | *Glyma.19G078300* | forward | 1014 | 6.54 | 36.4 |
| *GAPDH16* | *Glyma.19G106800* | reverse | 1212 | 8.42 | 43.1 |

**Table S2** Basic information of the sixteen soybean *GAPDH* genes.

**^1^**IDs are available in the soybean genome sequencing project database.

**^2^**ORF: open reading frame.
