# Supplementary figures and images for: Genome-wide identification and analysis of glyceraldehyde-3-phosphate dehydrogenase family reveals the role of GmGAPDH14 to improve salt tolerance in soybean (Glycine max L.)
Source: Front Plant Sci. 2023 Jun 6;14:1193044. doi: 10.3389/fpls.2023.1193044 (PMC10281054; doi:10.3389/fpls.2023.1193044)

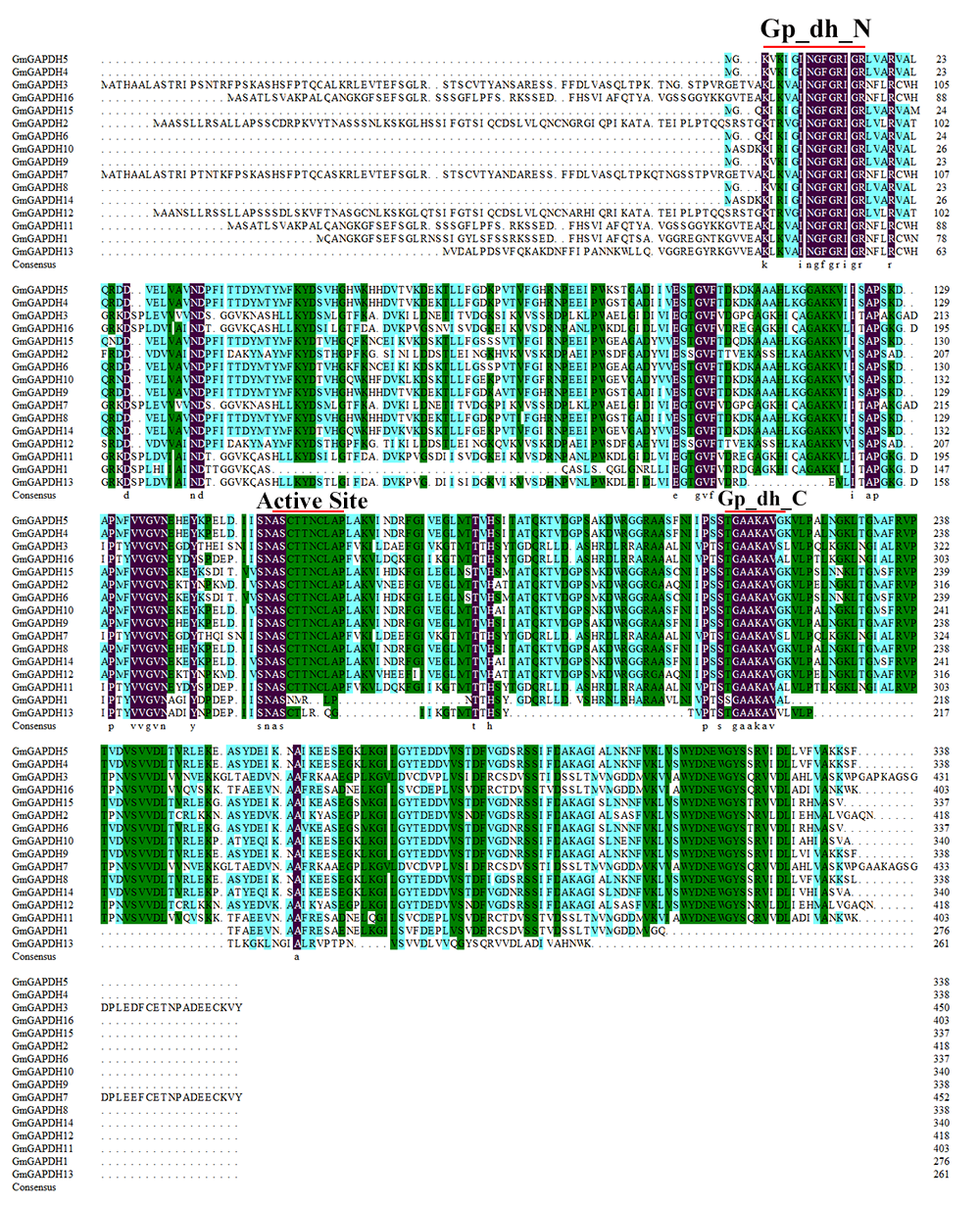

Supplement: Supplementary Figure 1 — Analysis of the conserved active site of GAPDH in soybean. [file DataSheet_1.zip › Supplementary Material/FigureS1.tif]

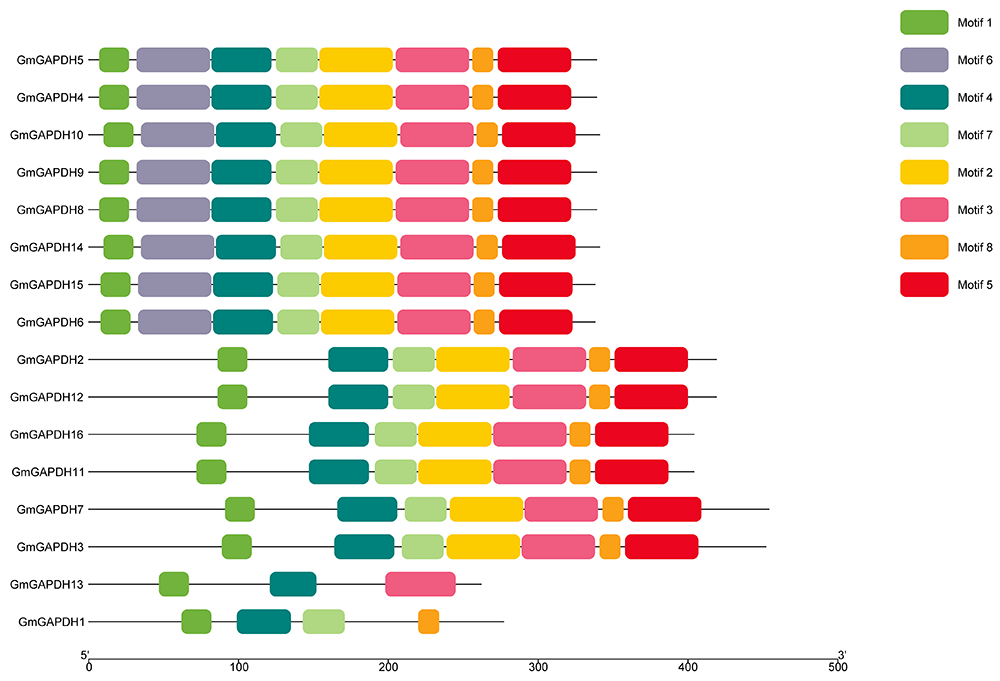

Supplement: Supplementary Figure 1 — Analysis of the conserved active site of GAPDH in soybean. [file DataSheet_1.zip › Supplementary Material/FigureS2.tif]

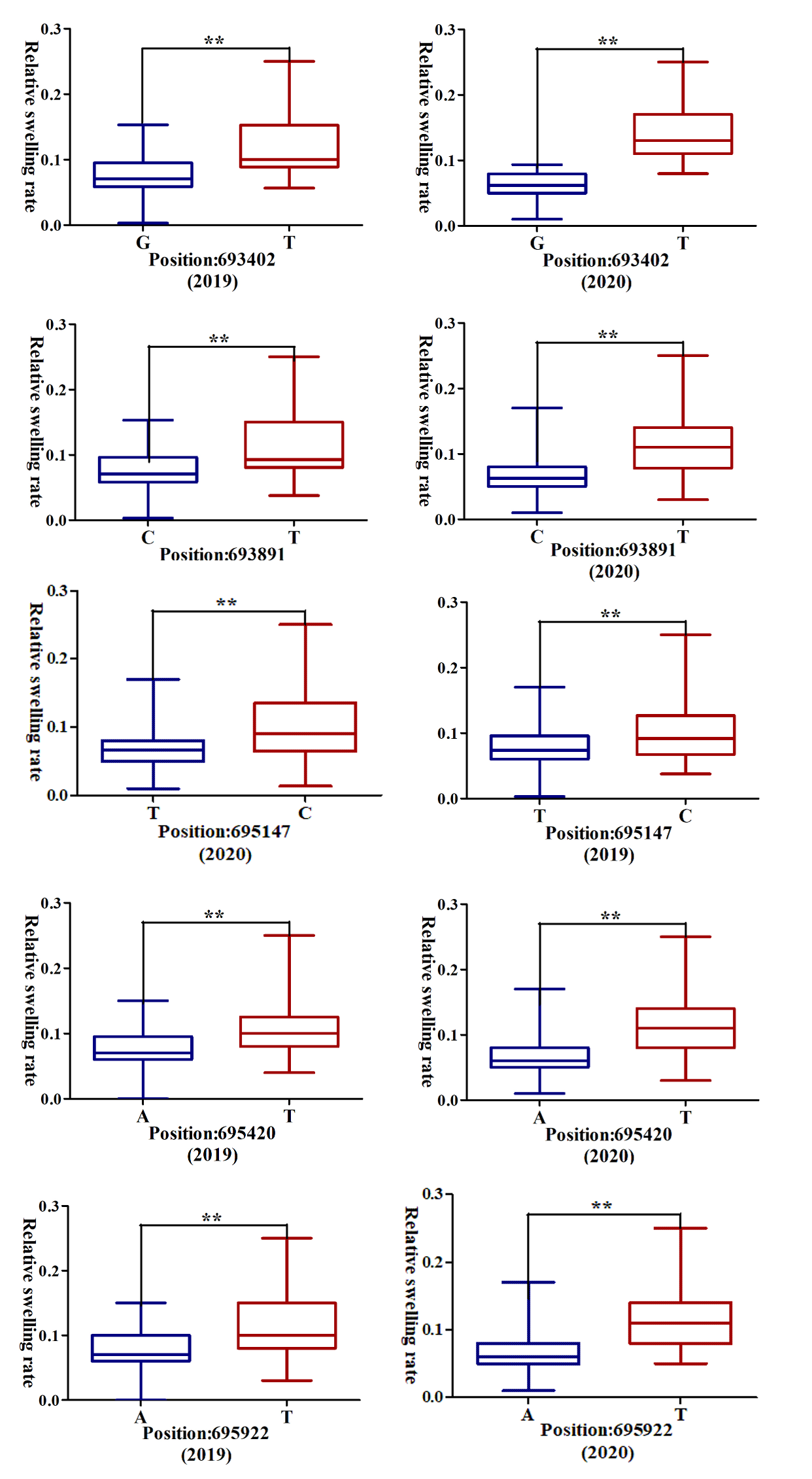

Supplement: Supplementary Figure 1 — Analysis of the conserved active site of GAPDH in soybean. [file DataSheet_1.zip › Supplementary Material/FigureS3.tif]
